# New Data on the Presence of Hemocyanin in Plecoptera: Recomposing a Puzzle

**DOI:** 10.1673/031.011.15301

**Published:** 2011-11-10

**Authors:** Valentina Amore, Brunella Gaetani, Maria Angeles Puig, Romolo Fochetti

**Affiliations:** ^1^ Environmental Sciences Department, University of Viterbo, 01100 Viterbo, Italy; ^2^ Centro de Estudios Avanzados de Blanes (CEAB-CSIC), 17300 Blanes, España

**Keywords:** cDNA, phylogeny, respiratory proteins, stoneflies

## Abstract

The specific role of hemocyanin in Plecoptera (stoneflies) is still not completely understood, since none of the hypotheses advanced have proven fully convincing. Previous data show that mRNA hemocyanin sequences are not present in all Plecoptera, and that hemocyanin does not seem to be uniformly distributed within the order. All species possess hexamerins, which are multifunction proteins that probably originated from hemocyanin. In order to obtain an increasingly detailed picture on the presence and distribution of hemocyanin across the order, this study presents new data regarding nymphs and adults of selected Plecoptera species. Results confirm that the hemocyanin expression differs among nymphs in the studied stonefly species. Even though previous studies have found hemocyanin in adults of two stonefly species it was not detected in the present study, even in species where nymphs show hemocyanin, suggesting that the physiological need of this protein can change during life cycle. The phylogenetic pattern obtained using hemocyanin sequences matches the accepted scheme of traditional phylogeny based on morphology, anatomy, and biology. It is remarkable to note that the hemocyanin conserved region acts like a phylogenetic molecular marker within Plecoptera.

## Introduction

The recent discovery of hemocyanin in many insect orders raises doubts about the common assumption that the tracheal system is sufficient for insect respiration, and that respiratory proteins are thus unnecessary. Our research is based on the first report of hemocyanin in the perlid stonefly *Perla marginata* (Hagner-Holler et al. 2004), and aims to better understand the presence, functional significance, and role of this protein in the Plecoptera (Fochetti et al. 2006; Amore et al. 2009; Amore and Fochetti 2009). A previous study assessed the presence/absence of hemocyanin mRNA in the larval and adult stage of chosen species belonging to the seven European stonefly families (Amore et al. 2009). Additionally, some selected Oriental and Afrotropical stonefly species living in rivers with different ecological features have been tested in respect to those in Palaearctic streams (Amore and Fochetti 2009; Amore et al. 2010). So far, we have investigated 33 species (present data included): 25 species belonging to the seven families of the two European superfamilies, five species belonging to Oriental Perlidae, one species of Oriental Peltoperlidae, and two species of African Notonemouridae. The target species was analyzed during different phases of the life cycle (nymphs and adults), and from various streams and river typologies (perennial temperate rivers, Mediterranean temporary streams, tropical rivers, high elevation rivers and lakes) (Amore and Fochetti 2009). Our data clearly show that mRNA hemocyanin sequences are not present in all Plecoptera (Fochetti et al. 2006; Amore et al. 2009; Amore and Fochetti 2009), and hemocyanin does not seem to be uniformly distributed within the order. All species possess hexamerins, which are multifunction proteins that probably originated from hemocyanin. We hypothesized that the presence of hemocyanin could depend on the length of the life cycle, body size, trophic role, or environmental induction. None of these hypotheses proved to be fully convincing (Amore et al. 2010), and the specific role of hemocyanin in Plecoptera is still not completely understood. However, by using liquid chromatography—tandem mass spectrometry, we proved that regardless of its putative function (respiratory, immune defense, storage protein), hemocyanin is actually expressed in species in which its mRNA is present (Amore et al. 2011). The hemocyanin expression pattern we have so far obtained for the entire Plecoptera order could also be explained by other functions besides respiration, but this investigation is beyond the scope of the present paper.

As far as nymphs are concerned, the present paper aims to extend the study on the presence/absence of this pigment to other Plecoptera genera/species that have not been investigated so far (the genera *Dyctiogenus, Perlodes, Besdolus, Arcynopteryx, Pachyleuctra*). In order to obtain an increasingly detailed picture of the hemocyanin presence and distribution across the order, and in an attempt to better understand functional significance and role of this protein in the Plecoptera, we studied Italian stenoendemisms (i.e., *Besdolus ravizzarum* Zwick and Weinzierl (1995)), Pyrenean endemics (i.e., *Pachyleuctra benllochi* (Navás 1917)), and species believed to have a very ancient origin, like the ercinic relict *Arcynopteryx compacta* (McLachlan, 1872).

In regards to adults, hemocyanin has only been recorded in *Perla marginata* (Hagner-Holler 2004) and *P. grandis* (Fochetti et al. 2006). In our previous studies we never detected hemocyanin in adults of other species (Amore and Fochetti 2009), even in species where we could sequence hemocyanin in nymphs, suggesting that the physiological request of hemocyanin can change during the life cycle. Here we extend the study to other species, to cover a representative sample of the European biodiversity of the order at the family level (genera *Pachyleuctra, Nemoura, Protonemura*).

## Materials and Methods

### Sequence analysis

Specimens belonging to the following ten species (nymphs and adults) in two families were collected and preserved in RNAlater (www.qiagen.com).

### Perlodidae

*Dyctiogenus alpinum* (Pictet, 1842) and *Perlodes intricatus* (Pictet, 1841) nymphs. Collected 1 February 2009. Po river, Pian della Regina, Crissolo, 1800 m (Cuneo—Piemonte Region, Italy).

*Besdolus ravizzarum* nymphs. Collected 3 February 2009. Curone stream, Val Curone 320 m (Alessandria—Piemonte Region, Italy). 44° 47′ 14″ N;9° 04′ 02″ E.

*Arcynopteryx compacta*, (McLachlan, 1872) nymphs. Collected 6 June 2009, Blue Lake, Rosellón, 2530 m. (Oriental Pyrenees Department, Languedoc Region, France) N 42,61554; E 1,96704.

*Isoperla acicularis* (Despax, 1936) ssp. *acicularis* nymphs and adults. Collected July 2008. Vallarties river, 1390 m. (Catalunya, Spain). 00° 48′ 10,9″ E; 42° 39′ 24,07″ N.

### Leuctridae

*Leuctra alosi* Navás, 1919. Adults. Collected July 2008. Vallarties river, 1390 m. (Catalunya, Spain). 00° 48′ 10,9″ E; 42° 39′ 24,07″ N.

*Pachyleuctra benllochi* (Navás, 1917). Nymphs and adults. Collected July 2008. Escita inlet, 1790 m. (Catalunya, Spain). 01° 00′ 56,0″ E; 42° 34′ 44,2″ N.

### Nemouridae

*Amphinemura sulcicollis* (Stephens, 1836). Adults. Collected July 2008. Vallarties tributary, 1390 m. (Catalunya, Spain). 00° 48′ 10,9″ E; 42° 39′ 24,07″ N.

*Nemoura cinerea* (Retzius, 1783), and *Protonemura tuberculata* Kempny, 1888. Adults. Collected July 2008. Peguera river and tributaries, 2295 m. (Catalunya, Spain). 01° 02′ 47,5″ E; 42° 32′ 43,9″ N.

Total RNA was extracted and degenerate oligonucleotide primers, designed according to hemocyanin conserved region (∼ 600 nucleotides), were used in a reverse transcriptase polymerase chain reaction. A β— actin fragment was used as control (*P. marginata* β—actin: HM991865, *B. ravizzarum* β—actin: HM991864). Polymerase chain reaction fragments of expected size were cloned into pGEM-T (Promega, www.promega.com) easy vector and sequenced by a commercial service as described in Amore et al. (2009). The sequences thus obtained were translated with the tool provided by ExPASy Molecular Biology Server of the Swiss Institute of Bioinformatics (www.expasy.org).

### Sequence data and multiple alignment

Two different multiple alignments of the proteins belonging to the hemocyanin superfamily (HcSF) were performed: the first one only for Plecoptera sequences, and the second for sequences of Plecoptera and other groups of arthropods.

**Multiple alignment: Plecoptera.** From our cDNA and from the Genbank database, sequences were deduced in 29 stonefly species of 14 hemocyanins (6 of the subunit 1 (hc1) and 8 of the subunit 2 (hc2)) and 27 hexamerins. Table 1 lists the sequences used for the alignment. Six Myriapoda hemocyanin sequences (i.e., *Scutigera coleoptrata* AJ344359, AJ344360, AJ431378, AJ431379, AJ512793 and *Spirostreptus* sp. AJ297738) were used in the alignment, since Myriapoda are in an ancestral position with respect to Plecoptera (Kusche and Burmester 2001). The final alignment included 48 sequences, 520 nucleotides, and 154 amino acids positions.

**Multiple alignment: Arthropod HcSF.** The alignment of Plecoptera sequences was completed with others sequences of the arthropod hemocyanin superfamily, retrieved from the GenBank database. The alignment was composed of crustacean prophenoloxidases (PPO), insect prophenoloxidases (PPO), crustacean cryptocyanins (CC) or pseudohemocyanins (Phc), crustacean hemocyanins (hc), Myriapoda hemocyanins (hc), insect hemocyanins (hc), and insect hexamerins (hx).

The hexamerin receptors were ignored in this study because only a small part of the sequences aligned well with the hemocyanin conserved region we analyzed. A list of sequences for the non—Plecopteran taxa used in this study is provided in Table 2. The final alignment comprises 102 sequences, 785 nucleotides, and 161 amino acid positions.

**Sequence alignment and phylogenetic inference.** Multiple alignment of nucleotides and amino acid sequences was constructed with the MAFFT online version (Katoh et al. 2005) matrix BLOSUM62. Long gap regions, as well as some highly divergent regions, were removed from the final data set, and in order to optimize the results of the phylogenetic analysis, we operated a selection of conserved blocks from multiple alignments with Gblocks server (Talavera and Castresana 2007). The appropriate phylogenetic model for nucletidic sequences was selected with MrModeltest2 (Nylander et al. 2004). The amino acid sequence evolution model was chosen by ProtTest (Abascal et al. 2005) using the Akaike information criterion. Nucleotidic tree constructions were performed by Bayesian analysis with MrBayes 3.1–2 (GTR model). The reliability of the trees was tested by bootstrap analysis (Felsenstein 1985) with 1000 replications. Amino acid trees were inferred by Maximum Likelihood (ML) methods. The phylogenetic analyses were performed with PhyML (www.atgcmontpellier.fr/phyml) (Guindon and Gascuel 2003) with100 replications. Distances between pairs of protein sequences were calculated according to the LG model (Le and Gascuel 2008) assuming a gamma distribution of substitution rate.

## Results

### Sequence analysis

The designed primers were applied to cDNAs reverse transcribed from the investigated species. When these primers were applied on nymphs, they produced fragments of the expected size. Two sequences were amplified for *D. alpinum* a.n. GU121395, GU121396, one sequence for *B. ravizzarum* a.n. GU121394, *A. compacta* a.n. GU121393, *P. intricatus* a.n. GU121397, *I. acicularis acicularis* a.n. GU121398, and *P. benllochi* a.n. GU121399. The amplified fragments were about 600 nucleotides long; the translated amino acid sequences resulted in about 195 amino acids, except for *P. intricatus*, that had an 893 nucleotides long amplified fragment and a translated sequence of 297 amino acids (Table 2). The same primers applied to adult specimens gave no band in PCR experiments.

**Figure 1.**
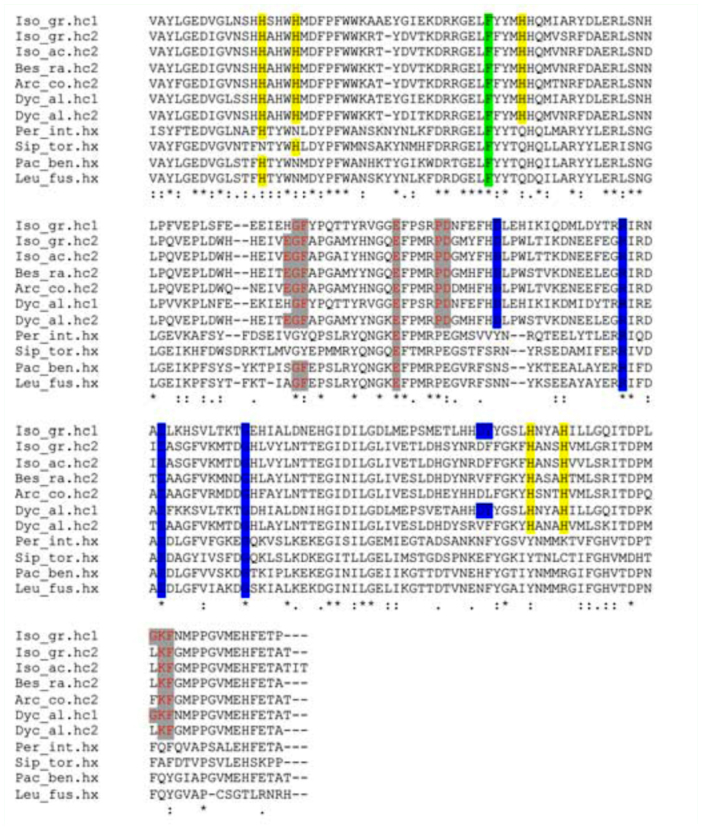
Multiple sequence alignment (BLOSUM62) of hemocyanins conserved amino acid sequences (hc) and correspondent hexamerins sequences (hx). His (yellow) and Phe (green) residues involved in the oxygen—binding site are indicated. The residues involved in the trimer (blue) and dimer (red) contacts are also shown. High quality figures are available online.

Both BLAST (Blastn and Blastp) and phylogenetic analyses (see below) unequivocally identified the sequences of *D. alpinum* (Dyc_al.hc1; Dyc_al.hc2), *B. ravizzarum* (Bes_ra.hc2), and *A. compacta* (Arc_co.hc2) as insect hemocyanins. The five histidines (His) of the studied fragment, crucial for the O_2_-binding function, were present in all subunits (Figure 1), while the sequence of *P. intricatus* (Per_in.hx) and *P. benllochi* (Pac_be.hx) were identified as hexamerins. In order to compare and describe the amino acid sequences of nymphs they were further compared to the hemocyanins known from the Perlodidae *Isoperla grammatica* (Iso_gr.hc1 and Iso.gr.hc2) (Amore et al. 2009), the Chloroperlidae *Siphonoperla torrentium* (Sip_tor.hx), and the Leuctridae *Leuctra fusca* (Leu_fus.hx) (Table 3). As expected, *D. alpinum* hemocyanin subunit 1 (Dyc_al.hc1) showed the highest degree of identity with the type 1 hemocyanin subunits (0.90 amino acidic and 0.85 nucleotidic), whereas lower scores were obtained when comparing type 2 subunits (0.53–0.55 amino acidic and 0.61–0.63 nucleotidic). Subunit 2 of *D. alpinum* (Dyc_al.hc2), *B. ravizzarum* (Bes_ra.hc2), *A. compacta* (Arc_co.hc2), *I. acicularis acicularis* (Iso_ac.hc2), and stonefly hemocyanin subunit 2 of *I. grammatica* displayed 0.86–0.95 identical amino acids, and 0.86–0.94 identical nucleotides, while lower identity scores were observed with other type 1 subunits (0.53–0.54 amino acidic and 0.60– 0.62 nucleotidic). Only one of the four Cubinding histidines is conserved in the *P. intricatus* and *P. benllochi* hexamerins. Comparison with hc1 and hc2 were in the range of 0.38–0.46 for amino acids and 0.53– 0.58 for nucleotides, whereas identity values were higher among Plecoptera hexamerins (0.58–0.83 amino acids and 0.69–0.86 nucleotides). *P. benllochi* showed a close relationship with *L. fusca* (0.83 amino acid and 0.86 nucleotide); both species belong to Leuctridae.

**Figure 2.**
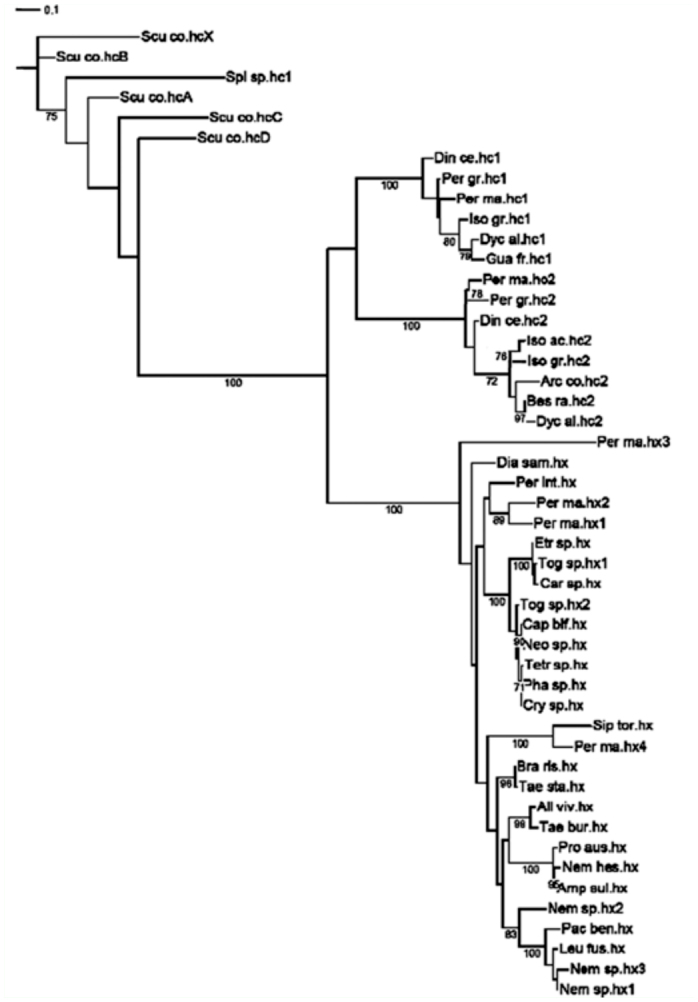
ML analysis of the Plecoptera hemocyanin superfamily (HcSF): hemocyanins (hc) and hexamerins (hx). The numbers represent the bootstrap support. The bar equals 0.1 substitutions per site. High quality figures are available online.

### Phylogenetic analysis

**Figure 3.**
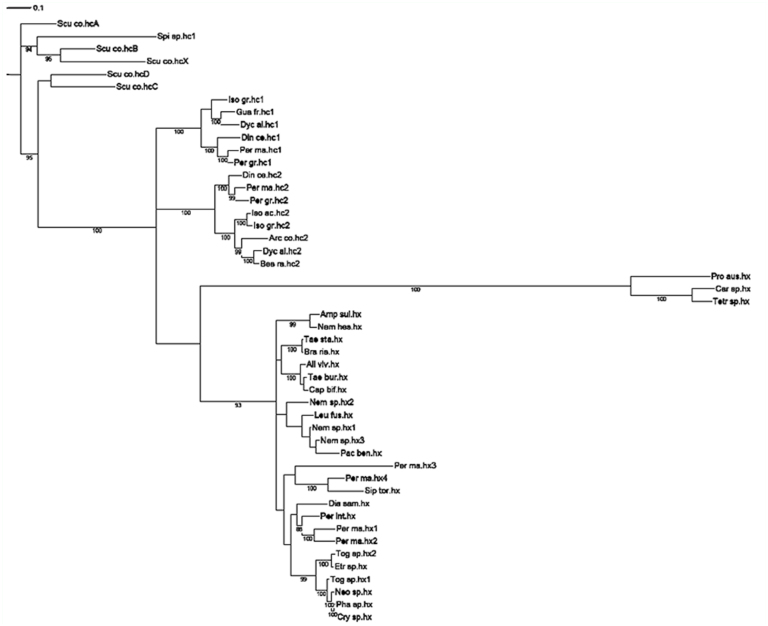
Bayesian analysis of the Plecoptera hemocyanin superfamily (HcSF): hemocyanins (hc) and hexamerins (hx). The numbers represent the bootstrap support. The bar equals 0.1 substitutions per site. High quality figures are available online.

Both types of analyses (Bayes and ML) gave similar tree topologies (Figures 2, 3).

### Plecoptera

The Myriapoda sequences were used to root the tree for graphics purposes. ML analysis (Figure 2) resulted in three well supported monophyletic clades. Dyc_al.hc1 joined the clade with the previously identified Plecoptera hemocyanin subunit 1 (100% bootstrap support) (Figure 2). Dyc_al.hc2, Bes_ra.hc2, Iso_ac.hc2, and Arc_co.hc2 grouped with hemocyanin subunit 2 (100% bootstrap support). Hexamerins, where Per_in.hx and Pac_be.hx grouped, formed a third clade (100% bootstrap support). Within hemocyanin subunit type 1, the Perlodidae sequences (Iso_gr.hc1; Guad_fr.hc1; Dyc_al.hc1) were monophyletic and derived from Perlidae (87% bootstrap support), whereas the Perlidae clade was more disordered. Within the clade of hemocyanin subunit 2, Perlidae and Perlodidae formed two distinct clades, even if the Perlodidae clade was supported by a 50% bootstrap value. The phylogenetic analysis indicated that the hemocyanin subunit 2 shares a common ancestor with all Plecoptera hexamerins. Within this clade the systematic relationship among groups were less resolved. The results of Bayesian inference (Figure 3) generate *a posteriori* distribution starting from an *a priori* probability: the Plecoptera tree shows an unresolved node when examining hc1, hc2, and hexamerin genetic affinity.

### HcSF

**Figure 4.**
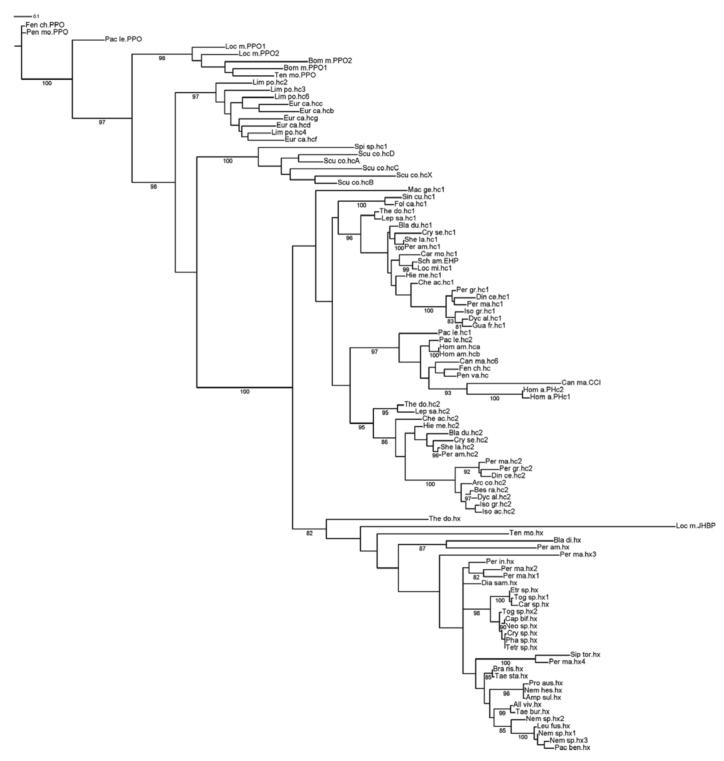
ML analysis of Arthropoda hemocyanin superfamily (HcSF): PPO, prophenoloxidases; hc, hemocyanins; hx, hexamerins; CCI and PHc cryptocyanins. The numbers represent the bootstrap support. The bar equals 0.1 substitutions per site. High quality figures are available online.

**Figure 5.**
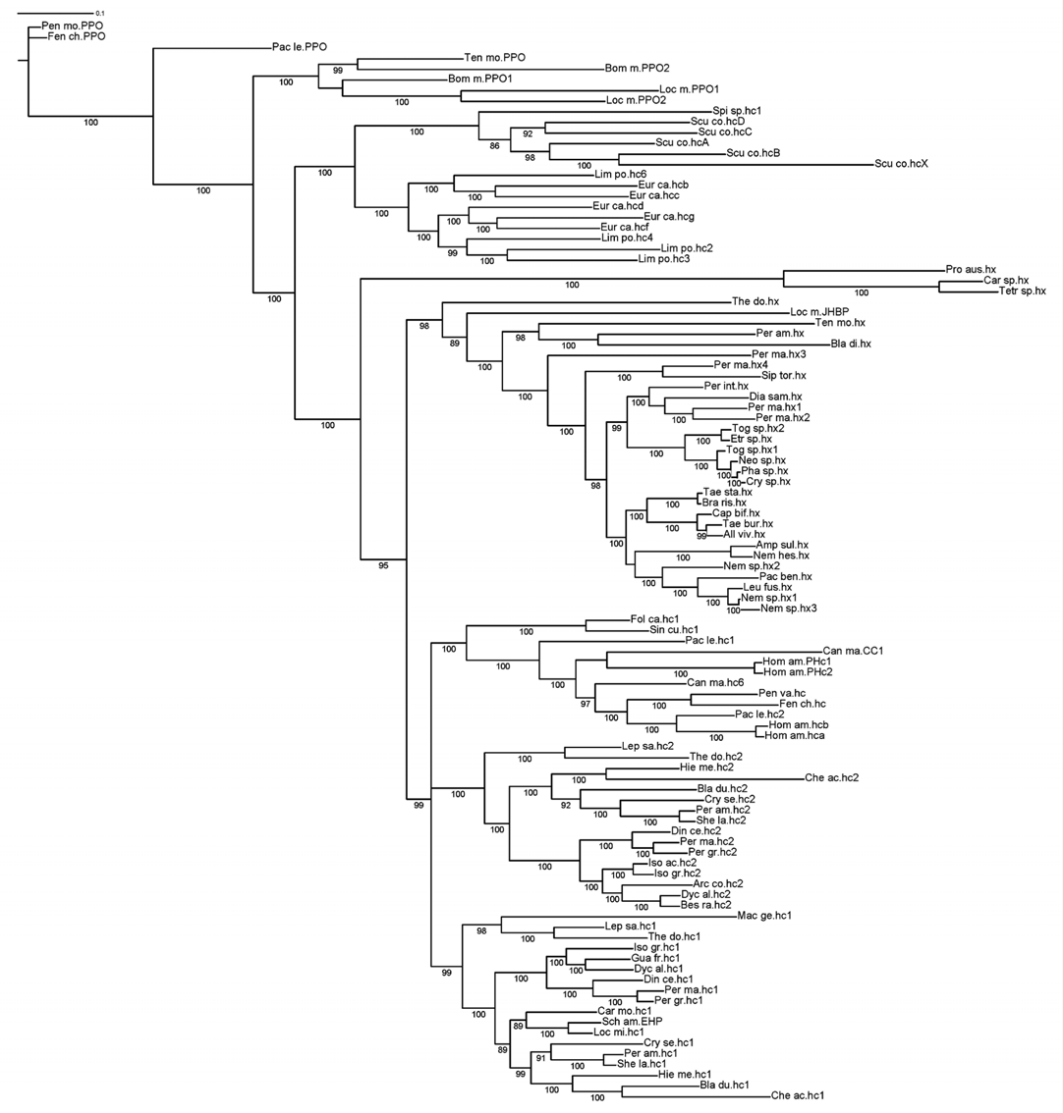
Bayesian analysis of the Arthropoda hemocyanin superfamily (HcSF). PPO, prophenoloxidases; hc, hemocyanins; hx, hexamerins; CCI and PHc cryptocyanins. The numbers represent the bootstrap support. The bar equals 0.1 substitutions per site. High quality figures are available online.

Within the hemocyanins, three distinct clades emerged in accordance with the phylogeny of arthropod subphyla. Both ML and Bayesian inference (Figures 4, 5) resulted in a branch representing Chelicerata hemocyanin (97– 100% bootstrap support), a second branch representing Myriapoda hemocyanin (100% bootstrap support), and a third branch for crustacean and insect hemocyanins, insect hexamerins, and crustacean cryptocyanins (95–100% bootstrap support). Myriapoda is the sister group with respect to crustacean and insect hemocyanins. All insect hexamerins formed a clade (82–98% bootstrap support). The results of ML analysis showed one clade for insect hemocyanin subunit 1, one clade for insect hemocyanin subunit 2, and one for crustacean hemocyanins and cryptocyanin. This macro—clade had a low bootstrap support (< 80%). At any rate, all hexapod hexamerins joined in the same clade in all analyses. In subunit 1, the hemocyanins from Zygenthoma (Ter_do.hc1 and Lep_sa.hc1) formed the sister group of the pterygote proteins (97% bootstrap support); Collembola (Sin_cu.hc1 and Fol_ca.hc1) was basal to the ectognathan subunits (38% bootstrap support). Within the hemocyanin subunit types 2, phylogeny resembled that of subunit types 1, and *Machilis germanica* (Mac_ge.hc1) was in an ambiguous position and clustered within hexamerins. In Bayesian inference (Figure 5), there was an unresolved node in the cluster including sequences of hexapod hc1 and hc2, crustacean hc, and cryprocyanin.

## Discussion

### Hemocyanin in nymphs

Our results suggest that the hemocyanin expression differs among nymphs of different stonefly species. The hemocyanin conserved region was sequenced in all nymphs, except in those of *P. intricatus* and *P. benllochi*, where only hexamerins were found. These results confirm that hemocyanin is not expressed in all Plecoptera species. It is worthy to note that *P. intricatus* and *D. alpinum* were collected in the same river, sampling site, and sampling date. Both species belong to Perlodidae, are medium—sized, semivoltine, and are mainly predators (Fochetti and Tierno de Figueroa 2008), but they display a different physiological response on hemocyanin production. On the other hand, *B. ravizzarum*, a Perlodidae living at lower altitude in the potamal river zone, expresses hemocyanin in its mRNA repertory.

Summarizing all the data regarding stoneflies published so far (Table 4) (Hagner-Holler et al. 2004; Fochetti et al. 2006; Amore et al. 2009; Amore and Fochetti 2009; Amore et al. 2010), we can confirm that hemocyanin expression in Plecoptera does not depend on size or trophic role. Environmental adaptation to ecological conditions might have led to the loss of the protein in some lineages. It is conceivable that independent adaptations to local conditions caused a decrease in hemocyanin requirement, a precondition to generate variability. Cumulative mutations and divergent evolution probably caused significant change in hemocyanin domain II to the point of disabling copper—binding sites and oxygen affinity, thus leading to ancestorlike hexamerin proteins.

### Hemocyanin in adults

Plecoptera are hemimetabolous insects whose habitat completely changes when they become adults. While nymphs live in aquatic habitats, adult stoneflies emerge from the streams, lakes, or rivers. They have reduced flight ability, and in some cases males are brachypterous (for the species investigated in the present paper, this condition occurs in *D. cephalotes* and *I. viridinervis*). They can generally be found on the banks next to the emergence area. Although the amount of oxygen in the air is much higher compared to the oxygen dissolved in water, it was proven that even insects that are terrestrial in all developmental phases possess respiratory proteins. In fact, hemoglobin genes were found in holometaboulos insects such as *Drosophila* (Hankeln et al. 2002) and *Apis* (Hankeln et al. 2006), as well as some Hemiptera, Coleoptera, and Lepidoptera that live in normoxic conditions (Burmester and Hankeln 2007).

Adults and nymphs have very different activities in the Plecoptera: the nymphal stage is mainly devoted to feeding and molting, thus undergoing considerable physiological stress (Fochetti and Tierno de Figueroa 2008; Tierno de Figueroa et al. 2003). Adults are mainly devoted to mating (Tierno de Figueroa et al. 2006), and in some cases they do not feed at all (for instance *Perla marginata, P. grandis, D. cephalotes*) (Tierno De Figueroa and Fochetti 2001).

Preliminary data on the presence of hemocyanin in adults was reported in Amore and Fochetti (2009). In the present study, the number of investigated species was extended to a representative of all the European families of the order. Hemocyanin had been previously recorded for *Perla marginata* (Hagner-Holler 2004) and *P. grandis* (Fochetti et al. 2006), but in our previous and present studies we never detected hemocyanin in adults, even in species where hemocyanin was sequenced in nymphs, suggesting that the physiological need of hemocyanin may change during the life cycle.

### Plecoptera hexamerins

Hexamerins were sequenced in nymphs and in the adult of *Capnia bifrons*, an ovoviviparous species (Hynes 1941; Fochetti and Tierno de Figueroa 2008). It is interesting to note that hexamerins are proteins usually expressed at high concentrations in larval and nymphal stages, though rarely seen in adults (Beintema et al. 1994). Insect hexamerins show significant similarities in structure and sequence to arthropod hemocyanins (Markl et al. 1992; Beintema et al. 1994; Burmester and Scheller 1996), and it has been suggested that hexamerins changed their function to storage proteins after losing the ability to bind oxygen (Markl and Winter 1989). Hexamerins serve mainly as sources of amino acids during nonfeeding periods, in larval molting or adult development (Telfer and Kunkel 1991; Haunerland 1996; Beintema et al. 1994), but can also function as carrier proteins for small organic compounds like steroid hormones, riboflavin and juvenile hormones (Enderle et al. 1983; Magee et al. 1994; Braun and Wyatt 1996), or may be involved in immune response (Hayakawa 1994; Beresford et al. 1997).

### Phylogenetic

**implications Plecoptera HcSF.** Starting from the hypothesis that a common ancestor of all modern Plecoptera possessed hemocyanin, this character was lost several times during the evolution of the order. A first loss might have happened in the Nemouroidea ancestor, since no hemocyanin was found in any of the Nemouroidea species analyzed in the present study. Second, hemocyanin might have been independently lost in some Perloidea lineages, such as in Chloroperlidae or in the genus *Perlodes*. This idea is in agreement with the accepted theory that, even if Plecoptera is a very ancient order (fossil stoneflies date from the early Permian), the existing families do not seem to be very old, and recent and repeated phenomena of speciation and extinction have been described (Zwick 2000). In species where we did not sequence hemocyanin, we only found hexamerins. Hexamerins evolved from hemocyanins in the early steps of insect evolution, so they are paralogous proteins. Our data would indicate that hexamerins evolved from subunit 2 (hc2), even though the analysis of a different dataset led Burmester and Hankeln (2007) to hypothesize hc1 as the probable closest subunit.

It is remarkable to note that the hemocyanin conserved region acts like a phylogenetic molecular marker within Plecoptera. Two branches of hemocyanin subunits (hc1 and hc2) are always evident in the topology of the trees, and the phylogenetic pattern obtained using hemocyanin conserved fragment matches the accepted scheme of traditional phylogeny based on morphology, anatomy, and biology even when examining taxonomy of subfamilies (e.g. Perlodinae, Isoperlinae, and Arcynopteryginae within Perlodidae). Hexamerins follow more loosely the accepted systematic arrangement, indicating a lower evolutionary pressure that allowed them to accumulate mutations and distinct types of amino acids (Telfer and Kunkel 1991; Burmester et al. 1998). The use of hemocyanin as a molecular marker could be interesting to study in detail taxa whose systematic position within Plecoptera in still uncertain—such as the relationships between Perlidae, Perlodidae and Chloroperlidae—and to analyze phenomena of speciation and adaptation.

### Arthropoda HcFS

Chelicerata hemocyanins form a separate clade. A phenoloxidase activity of some subunit of chelicerata hemocyanin has been noted (Decker et al. 2001); therefore, these subunit types may be considered as transitional structures between phenoloxidases and hemocyanins.

The Myriapoda hemocyanins clade is the sister group of insect and crustacean hemocyanin and their derivates (insect hexamerins and crustacean cryptocyanins) according to Kusche and Burmester (2001). Assuming that protein phylogeny reflects species evolution, the presence of a unique clade for crustacean and hexapod hemocyanins and descendents strongly supports the Pancrustacea hypothesis, where all crustaceans and hexapods are included in a unique monophyletic taxon, in contrast to the Atelocerata hypothesis in which Myriapoda and Hexapoda are sister taxa, and Crustacea are more distantly related (see Brusca and Brusca 2002).

Hexamerins and cryptocyanins underwent parallel evolution. The hexamerins form a monophyletic clade, which is the sister group of the known insect and crustacean hemocyanin, while cryptocyanins derived from crustacean hemocyanins (Beintema et al. 1994; Burmester and Scheller 1996; Durstewitz and Terwillinger 1997; Burmester et al. 1998; Burmester, 1999a, 1999b, 2001; Pick et al. 2009).

### Further considerations

The study of hemocyanin in insects is at the center of an ongoing scientific debate. Several studies have explored the functional properties of Arthropod hemocyanins and have led to a plethora of hypothetical functions, which include its role as an oxygen carrier (Markl et al. 1979a, 1979b; Markl and Decker 1992), or its non—respiratory functions having phenoloxidase and antimicrobial activity (Terwilliger 1998; Bridges 2001; Decker and Jaenicke 2004; Jaenicke and Decker 2004).

Recent studies on chelicerates, which have no phenoloxidases, stressed that evolution has developed a double function for this molecule, suggesting that hemocyanin acquires a phenoloxidase activity after proteolitic cleavage at the amino—terminal part (Decker and Rimbke 1998, Decker and Tuczek 2000). Hemocyanins of *Eurypelma californicum, Limulus polyphemus,* and *Tachypleus tridentatus* are comparable to phenoloxidases based on activation mechanisms, substrate specificity, and inhibition (Nagai and Kawabata 2000; Nagai et al. 2001).

The role of hemocyanins in immune response seems to be present in chelicerates and also in crustaceans. Under normal conditions the hemocyanin functions as an oxygen carrier, but it may be converted to phenoloxidase after microbial infections. In some Crustacea (*Penaeus vannamei* and *P. stylirostris*), antimicrobial and antifungal peptides can be cleaved from the C—terminal domain of hemocyanin (Destoumieux-Garzó et al. 2001; Lee et al. 2003) Additionally, hemocyanin concentration is associated with the molting cycle, suggesting a specific utilization during starvation (Depledge and Bjeregaard 1989). Under special circumstances, hemocyanin is metabolically recycled and employed as a source of energy from amino acids (Zuckerkandl 1960; Hagerman 1986). What remains to be determined is if hemocyanin has functions other than respiration in the Hexapoda. *In vitro* or *in vivo* studies on functions other than respiration have not yet been carried out in Hexapoda.

### Open questions

The present study focuses on Arctoperlaria species (the Northern hemisphere Plecoptera suborder) mainly on European fauna. The only sequence of the Antarctoperlaria (the Southern hemisphere Plecoptera suborder), *Dhiamphipnopsis samali*, included in our phylogenetic analysis derives from a specific study on Plecoptera hexamerin (Hagner-Holler et al. 2007). Enlarging the study to Antactoperlaria would give a wider general indication to the problematic investigation of hemocyanin distribution in Plecoptera.

Another issue concerns the plasticity of hemocyanin with respect to environmental context. Changes in hemocyanin expression can affect the total concentration of hemocyanin in the hemolymph or can modify the level of expression of a single subunit with respect to the others. Experiments aimed to monitoring adaptive physiology of Plecoptera in response to environmental stimuli, at the level of protein expression modulation and subunit ratio, are in progress with quantitative real—time PCR. If oxygen affinity and cooperativity of hemocyanin, and consequently the capacity of oxygentransport, are adapted to environmental conditions, then possessing hemocyanin represents a potential adaptive capacity for animals in the context of global warming. In this future context, the presence of hemocyanin and its variability in subunits type and multimeric formation may represent a focal aspect to be analyzed from the perspective of ecological selection (Schluter 2001).

**Table 1.**
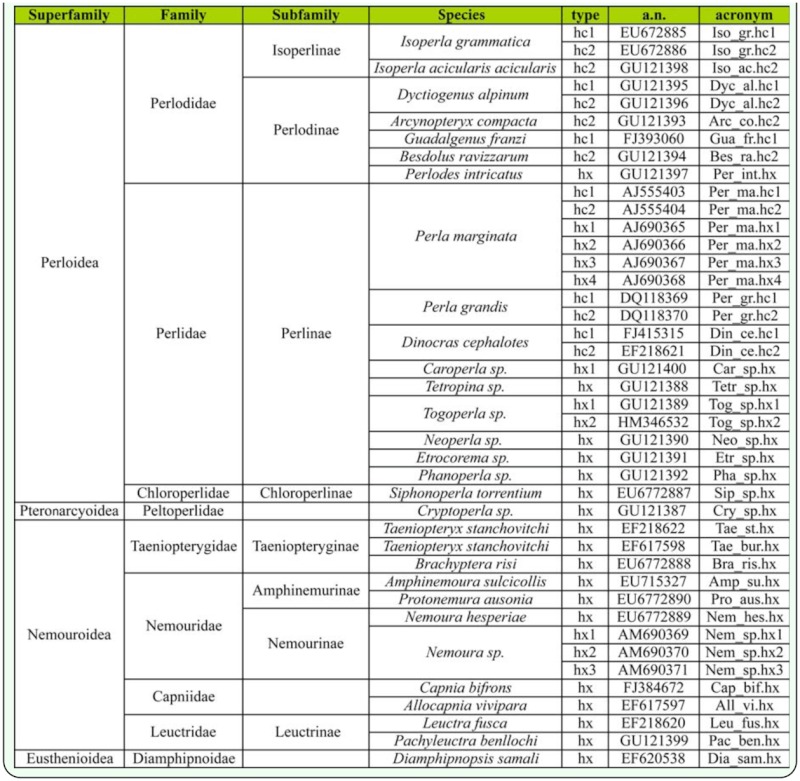
List of stoneflies species included in phylogenetic analysis, showing acronyms and GenBank accession number (a.n.).

**Table 2.**
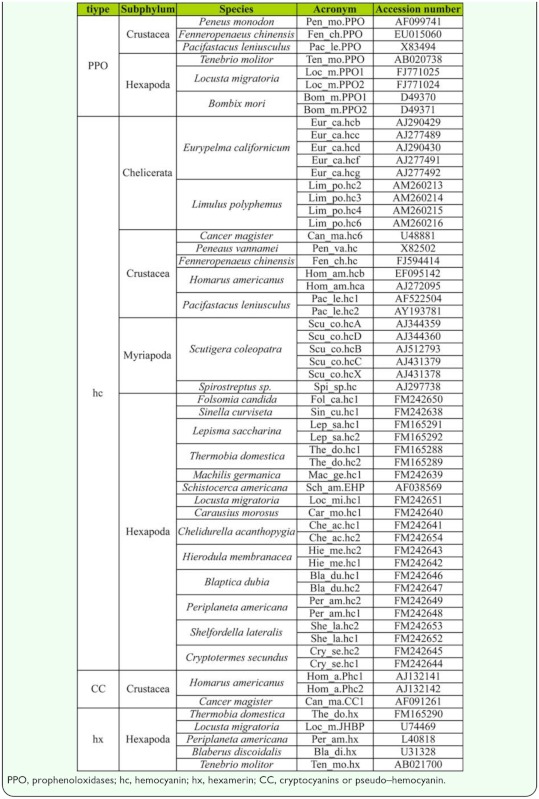
List of arthropod species, other than Plecoptera, included in the Plecoptera and Arthropod HcSF multiple alignment. Protein type, systematic position (subphylum and species), and GenBank accession number are shown.

**Table 3.**
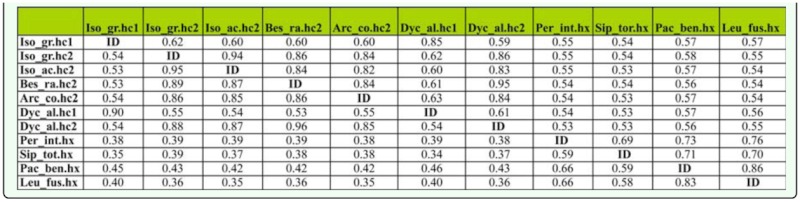
Nucleotidic (above) and amino acidic (below) identity. Species acronyms are as in the phylogenetic analysis. Seq: sequences.

**Table 4.**
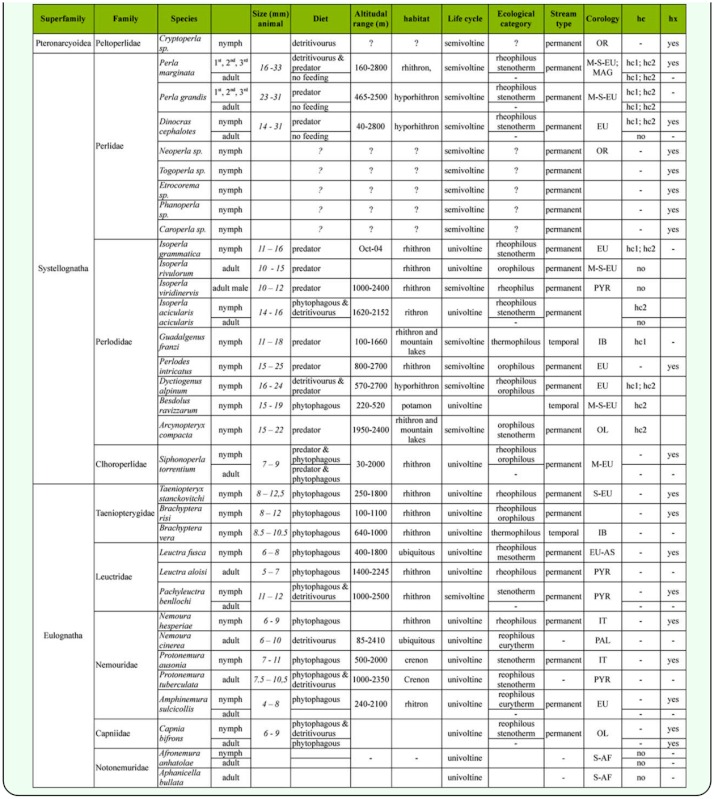
Systematic position, autoecology (size, trophic role, altitudinal range, habitat type and stream type, ecological category, corology), hemocyanin (hc), and hexamerins (hx) presence in nymph and adult stage of studied species. AF, Africa; PAL, Palaeartic; PYR, Pyrenees; EU, Europe; EU–AS, euroasiatic; OL, Oloartic; OR, Oriental; MAG, Maghreb; M, medium; S, South; N, North.

## Abbreviations:

HcSF: hemocyanin superfamily
